# Integrated Metabolo-Transcriptomics Reveals Fusarium Head Blight Candidate Resistance Genes in Wheat QTL-Fhb2

**DOI:** 10.1371/journal.pone.0155851

**Published:** 2016-05-27

**Authors:** Dhananjay Dhokane, Shailesh Karre, Ajjamada C. Kushalappa, Curt McCartney

**Affiliations:** 1 Department of Plant Science, Macdonald Campus, McGill University, 21,111 Lakeshore Road, Sainte-Anne-de-Bellevue, Quebec, H9X 3V9, Canada; 2 Agriculture and Agri-Food Canada, 195 Dafoe Road, Winnipeg, Manitoba, R3T 2M9, Canada; The University of Wisconsin - Madison, UNITED STATES

## Abstract

**Background:**

Fusarium head blight (FHB) caused by *Fusarium graminearum* not only causes severe losses in yield, but also reduces quality of wheat grain by accumulating mycotoxins. Breeding for host plant resistance is considered as the best strategy to manage FHB. Resistance in wheat to FHB is quantitative in nature, involving cumulative effects of many genes governing resistance. The poor understanding of genetics and lack of precise phenotyping has hindered the development of FHB resistant cultivars. Though more than 100 QTLs imparting FHB resistance have been reported, none discovered the specific genes localized within the QTL region, nor the underlying mechanisms of resistance.

**Findings:**

In our study recombinant inbred lines (RILs) carrying resistant (R-RIL) and susceptible (S-RIL) alleles of QTL-Fhb2 were subjected to metabolome and transcriptome profiling to discover the candidate genes. Metabolome profiling detected a higher abundance of metabolites belonging to phenylpropanoid, lignin, glycerophospholipid, flavonoid, fatty acid, and terpenoid biosynthetic pathways in R-RIL than in S-RIL. Transcriptome analysis revealed up-regulation of several receptor kinases, transcription factors, signaling, mycotoxin detoxification and resistance related genes. The dissection of QTL-Fhb2 using flanking marker sequences, integrating metabolomic and transcriptomic datasets, identified 4-Coumarate: CoA ligase (*4CL*), callose synthase (*CS*), basic Helix Loop Helix (*bHLH041*) transcription factor, glutathione S-transferase (*GST*), ABC transporter-4 (*ABC4*) and cinnamyl alcohol dehydrogenase (*CAD*) as putative resistance genes localized within the QTL-Fhb2 region.

**Conclusion:**

Some of the identified genes within the QTL region are associated with structural resistance through cell wall reinforcement, reducing the spread of pathogen through rachis within a spike and few other genes that detoxify DON, the virulence factor, thus eventually reducing disease severity. In conclusion, we report that the wheat resistance QTL-Fhb2 is associated with high rachis resistance through additive resistance effects of genes, based on cell wall enforcement and detoxification of DON. Following further functional characterization and validation, these resistance genes can be used to replace the genes in susceptible commercial cultivars, if nonfunctional, based on genome editing to improve FHB resistance.

## Introduction

Crop plants in natural environment significantly suffer from several devastating diseases, leading to severe economic losses. Plants resist pathogens through both constitutive or pre-existing and induced or *de-novo* synthesized metabolites and proteins, following pathogen invasion [1]. These resistance metabolites and proteins may be either structural or biochemical. The constitutive structural barriers include thick cuticles [2] and cell walls [3], and the resistance biochemicals include preformed antimicrobial or toxic secondary metabolites and proteins called phytoanticipins [1,4]. Phytoanticipins are constitutively synthesized secondary plant metabolites that provide defense at the outer layers in plants. These may be stored in nontoxic forms, but are released in active forms upon pathogen attack, with simple hydrolysis, as antimicrobial compounds [5]. The induced biochemicals, also known as phytoalexins, include hundreds of resistance metabolites, monomers and polymers, produced following pathogen invasion [6,7]. The induced biochemicals can also be proteins, also known as pathogenesis related (PR) proteins [3,8]. The induced structural barriers include formation and deposition of cell wall enforcing compounds such as hydroxycinnamic acid amides (HCAAs) which contain the pathogen to initial infection site [6].

Resistance in plants against pathogen attack has been considered to be qualitative or hypersensitive response, and quantitative or reduced susceptibility [9, 10]. But the distinction between them are not always clear, rather they are shades of gray [11]. Recently a novel unifying concept of resistance has been proposed and the resistance has been defined as the reduced susceptibility. The resistance is controlled by hierarchies of *R* genes, with regulatory and resistance related (RR) metabolite and protein biosynthetic roles [12]. The resistance is mainly due to resistance related (RR) metabolites and RR proteins, due to their antimicrobial or cell wall reinforcement properties. These RR metabolites and RR proteins can be constitutive (RRC) or induced (RRI) [13]. The pathogens, following inoculation, produce elicitors that are recognized by the membrane localized receptors called elicitor recognition receptors (ELRRs), encoded by *R*_*ELRR*_ genes, which then trigger downstream genes to induce elicitor triggered immunity (ELTI), the first line of defense [9–12]. Specialized pathogens enter into the cell, produce effectors by avirulence (AVR) genes, which in turn are recognized by the host effector recognition receptors (ERRs), encoded by *R*_*ERR*_ genes, to induce effector triggered immunity (ETI), the second line of defense [9–12]. Both ELTI and ETI result in hypersensitive response, thus considered to be qualitative resistance [12]. The ETI is considered to be due to *R*_*ERR*_ genes, and because of simple inheritance it has been extensively used in developing resistant plants. However, this resistance often breaks down because these genes are only receptor genes, and thus, their association with downstream resistance genes must be confirmed in a given cultivar [12,14]. The resistance is mainly due to RR metabolites and RR proteins, which are either constitutively produced (RRC) or induced (RRI), following pathogen invasion [13].

Wheat [*Triticum aestivum* L. (2n = 6x = 42)] is the second most important cereal crop with multi-utilitarian value, feeding 40% of the world’s population. Fusarium head blight (FHB) caused by *Fusarium graminearum* Schwabe [telomorph: *Gibberella zeae* Schw. (Petch)] is one of the most devastating and alarming diseases of wheat ruining harvests, in many wheat producing regions of the world, including Canada [15,16]. The accumulation of mycotoxins such as deoxynivalenol (DON) and nivalenol (NIV) is of major concern due to their detrimental effects on humans and animals [17]. The development of FHB resistant cultivars is considered to be the best way to manage this disease and the accumulation of mycotoxins in grains, as it is the most efficient, economic, and ecofriendly approach to manage FHB [15].

FHB resistance is quantitative in nature, involving several genes, each with small or large effects, and the phenotype is the result of their additive effects. Three different types of FHB resistance in wheat have been recognized and used in breeding: (i) resistance to initial infection or spikelet resistance (type-I), (ii) resistance to spread within the spike or rachis resistance (type-II), and (iii) resistance to mycotoxin accumulation in grains (type-III) [18]. The development of resistant cultivars is very challenging because of limited understanding of genetics of resistance and lack of cost-effective means to phenotype [19]. The screening is generally done based on spray inoculation which leads to high experimental errors, leading to inconsistent ranking of genotypes over years. Inoculation under controlled conditions can significantly reduce the experimental error and can enable quantification of both spikelet and rachis resistance [20].

More than 100 quantitative trait loci (QTLs) for FHB resistance have been identified in wheat [21]. The FHB resistant QTLs, with major and/or minor effects, have been mapped on all the wheat chromosomes, except on 7D [21]. Major QTLs on chromosome 3B (QTL-Fhb1) [22], 6B (QTL-Fhb2) [23] and 2D (QTL-2DL) [16], exhibit rachis resistance, and on chromosomes 5A (QTL-Fhb5) [22] and 4B (QTL-Fhb4) [16] confer spikelet resistance. The transfer of resistant QTLs based on marker assisted breeding is not very practical because these QTLs, in general, contain undesirable genes that are also transferred due to linkage drag.

The QTL-Fhb2 localized on the short arm of chromosome 6B is the second major QTL conferring rachis resistance [23]. The QTL-Fhb2 has been mapped as a Mendelian factor, spanning a region of 4.2 cM flanked by two simple sequence repeat (SSR) markers, GWM-133 and GWM-644, using recombinant inbred (RIL) population derived from a cross between BW278 (resistant parent) and AC foremost (susceptible parent), and the QTL explained 24.1% of resistance to FHB [23]. Resistant parent BW278 is a descendant of Sumai-3, a Chinese bread wheat cultivar which possesses high level of rachis resistance [22]. Similarly, the QTL-Fhb2 was mapped on 6BS using double haploid population, which conferred 21% resistance to FHB [24]. Both the studies reported high levels of rachis resistance, but no study has reported the specific genes localized in the QTL-Fhb2 region conferring resistance, nor the mechanisms of resistance. As the QTL regions contain several genes, the dissection and functional characterization of each gene localized in the QTL region on genomic scale is a very challenging task, especially in wheat which possesses a highly complex genome and lacks a complete genome sequence [25]. Therefore, genes in the QTL regions and the resistance mechanisms governed by them are not studied in detail. Even though significant attempts have been made to identify candidate genes localized within the QTL-Fhb1 [6,26], QTL-Fhb5 [27], so far no FHB resistance gene has been identified and validated.

Technological advancements in genome sequencing and integration of omics platforms have offered novel insights to explore the regulation of metabolic pathways and their biosynthetic genes underlying disease resistance mechanisms [6,28–30]. Metabolomics is a potential post-genomics tool to elucidate the host biochemical responses under biotic stress, to identify the candidate *R* genes, and to validate gene functions [6,31–33]. Non-targeted metabolomics has been applied to reveal the host biochemical mechanisms of quantitative resistance in crop plants such as wheat [6,34], and barley against *F*. *graminearum* [35–38], and potato against *Phytophthora infestans* [28–30,33]. Non-targeted metabolic profiling of wheat near isogenic lines (NILs) with FHB resistant QTL-Fhb1 revealed deposition of HCCAs in the cell wall that reduced further spread of pathogen within rachis, thus imparting resistance [6]. Higher abundance of several resistance metabolites belonging to phenylpropanoids, flavonoids, fatty acids, and terpenoids that inhibited the growth of *F*. *graminearum* and trichothecene biosynthesis was identified in barley against *F*. *graminearum* [35,38]. Metabolic profiling of resistant and susceptible potato cultivars against late blight identified phenylpropanoids and their biosynthetic genes regulated by *StWRKY1* [28].

The transcriptome is highly active and instantly changes with response to cellular perturbations. The study of wheat transcriptome under *Fusarium* stress revealed the expressed genes following FHB infection [39,40]. QTL-Fhb1 specific RNA-seq of Wangshuibai and its mutant NAUH117 (lacking a chromosome region including QTL-Fhb1 segment), revealed association of *PR5*, *PR14*, ABC transporter and jasmonic acid pathway genes in FHB resistance in wheat [39]. Transcriptome analysis using RNA-seq in maize upon *Fusarium verticillioides* inoculation revealed the involvement of shikimate, flavonoid, lignin and terpenoid biosynthetic pathways in imparting FHB resistance [40]. A lipid transfer protein (*LTP*) was found to be constitutively more abundant in NIL carrying QTL-Fhb5, based on microarray [27]. QTL-specific microarray analysis of Sumai-3 and two susceptible NILs showed up-regulation of 25 genes and the genes encoding PR proteins, like β-1-3 glucanase (*PR-2*), thaumatin like proteins (*PR-5*) and wheatwins (*PR-4*) were significantly over-expressed in genotypes containing Sumai-3 3BS region [41]. Microarray analysis of near isogenic lines carrying QTL-3BS, showed the up-regulation of genes involved in cell wall biogenesis upon fusarium infection [42]. A gene UDP-glycosyltransferase was reported to be highly over-expressed in NILs harboring two QTL-Fhb1 and QTL-Fhb5, based on microarray analysis, which has a major role in the detoxification of deoxynivalenol [42].

Considering this background, RILs carrying resistant and susceptible alleles of genes in QTL-Fhb2 were subjected to metabolome and transcriptome profiling upon *F*. *graminearum* inoculation. Our study is the first report that revealed six putative resistance genes localized within the QTL-Fhb2 region and also the plausible association of cell wall enforcing metabolites, explaining the underlying mechanisms of resistance, based on the integration of metabolomics and transcriptomics. The application of these genes, following validation, is discussed.

## Results

### Disease severity of RILs

**Fig 1:**
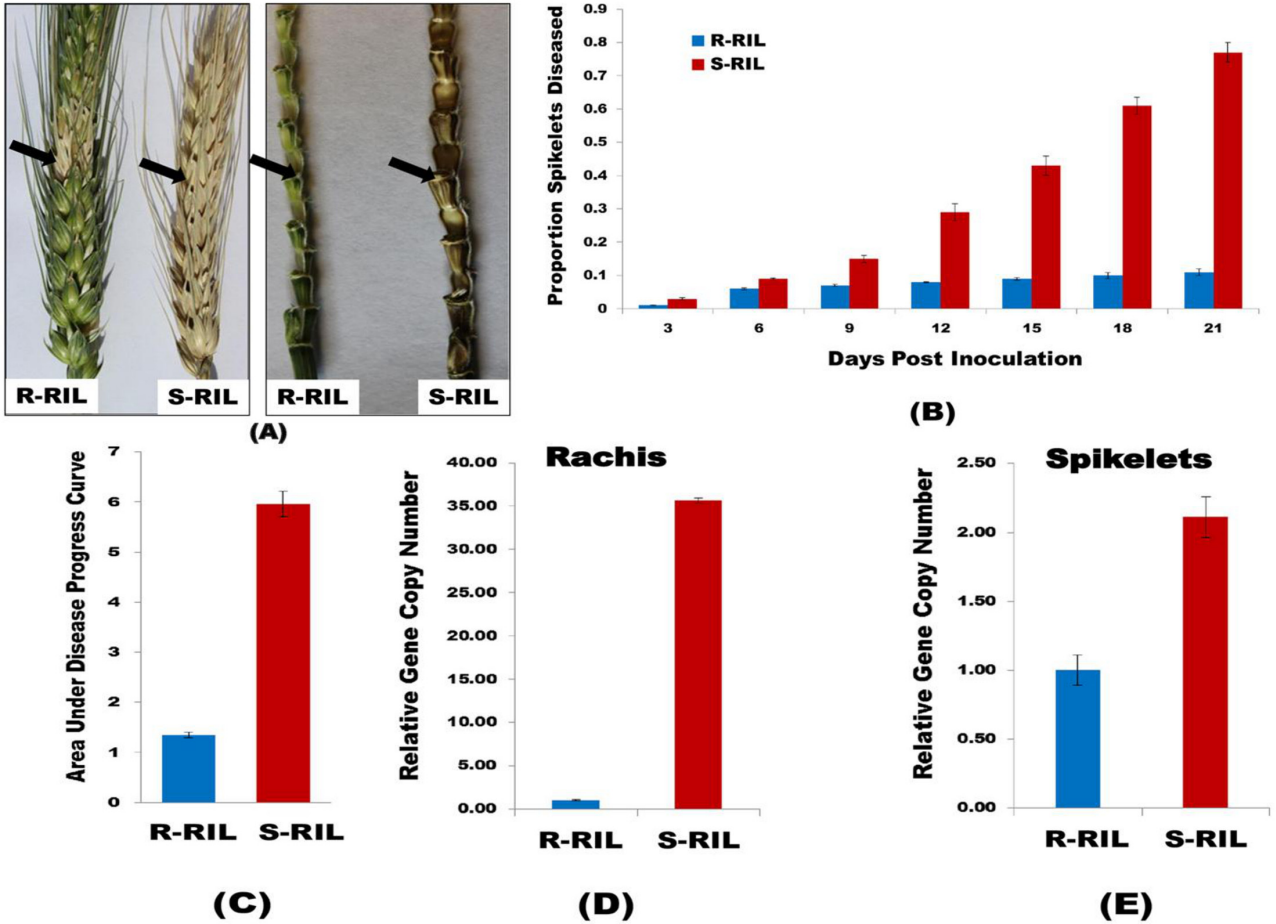
Phenotyping of RILs. (A). Spike and rachis of R-RIL and S-RIL, 21 dpi with *F*. *graminearum* spore suspension. A single alternate pair of spikelets in a spike was inoculated; black arrows indicate the site of inoculation. The spike and rachis in R-RIL shows only necrotic spots or diseased symptoms limited to the inoculated spikelet, while in S-RIL both spikelet and rachis are entirely diseased. (B). Proportion of Spikelets Diseased (PSD). A single pair of spikelets of a spike was inoculated in both the RILs and the proportion of spikelets diseased was recorded at 3 days intervals until 21 days, from which the PSD was calculated. The bar graph shows high PSD in S-RIL as compared to R-RIL. (C). The bar graph shows area under disease progress curve (AUDPC) calculated from PSD, significantly higher in S-RIL. (D) and (E). Fungal Biomass quantification in RILs. Three alternate pair of spikelets were inoculated with *F*. *graminearum* spore suspension and samples were collected at 7 dpi. The total genomic DNA was extracted and the relative gene copy number of *Tri6* was estimated using 2^−ΔΔC^_T_ method. (D). Shows the relative gene copy number of *Tri6* in rachis tissues; (E). Shows the relative gene copy number of *Tri6* in spikelet tissues. In both graphs, the gene copy number of *Tri6* is significantly higher in S-RIL as compared to R-RIL.

Spikelets showing necrotic lesions and bleaching symptoms were considered as diseased. The diseased symptoms were visible at 3 dpi as small, tiny necrotic spots on the inoculated pair of spikelets. The numbers of spikelets diseased in R-RIL were very few; the fungus was able to colonize only spikelets adjacent to the inoculated pair of spikelet, not further, even at 21 dpi. On other hand, in S-RIL the fungus was able to spread through rachis making the whole spike diseased at 21 dpi (Fig 1A). The rachis was lush green in R-RIL without any disease symptoms in rest of the spikelets, unlike in S-RIL where both rachis and spikelets were diseased showing blackish brown discoloration with necrotic lesions. This clearly demonstrated that the QTL imparts high rachis resistance as the fungus was unable to spread through rachis in R-RIL. The PSD in S-RIL was highly significant than in R-RIL (Fig 1B). The AUDPC calculated from PSD was significantly higher (*P* < 0.001) in S-RIL (AUDPC = 5.95) than in R-RIL (AUDPC = 1.34) (Fig 1C).

### Quantity of fungal biomass in RILs

The quantity of fungal biomass, quantified as relative copy number of *Tri6* gene, was highly significant in rachis of S-RIL as compared to R-RIL (Fig 1D). The reduced amount of fungal biomass in rachis of R-RIL, due to reduced ability of the pathogen to colonize the rachis, clearly demonstrates the role of QTL-Fhb2 in the R-RIL. Whereas, in the S-RIL the *Tri6* gene copy number was high, as the fungus was able to move through the rachis to the adjacent spikelets, meaning absence of rachis resistance. The relative gene copy number of *Tri6* was also calculated for the spikelets collected from both R-RIL and S-RIL, and it was found that the *Tri6* gene copy number was also significantly higher in S-RIL than in R-RIL, but the fold change was comparatively lower in spikelets than in rachis (Fig 1E). These results clearly demonstrate that the resistant alleles of genes in QTL-Fhb2 impart high rachis resistance.

### Resistance related induced (RRI) metabolites associated with QTL-Fhb2

**Fig 2:**
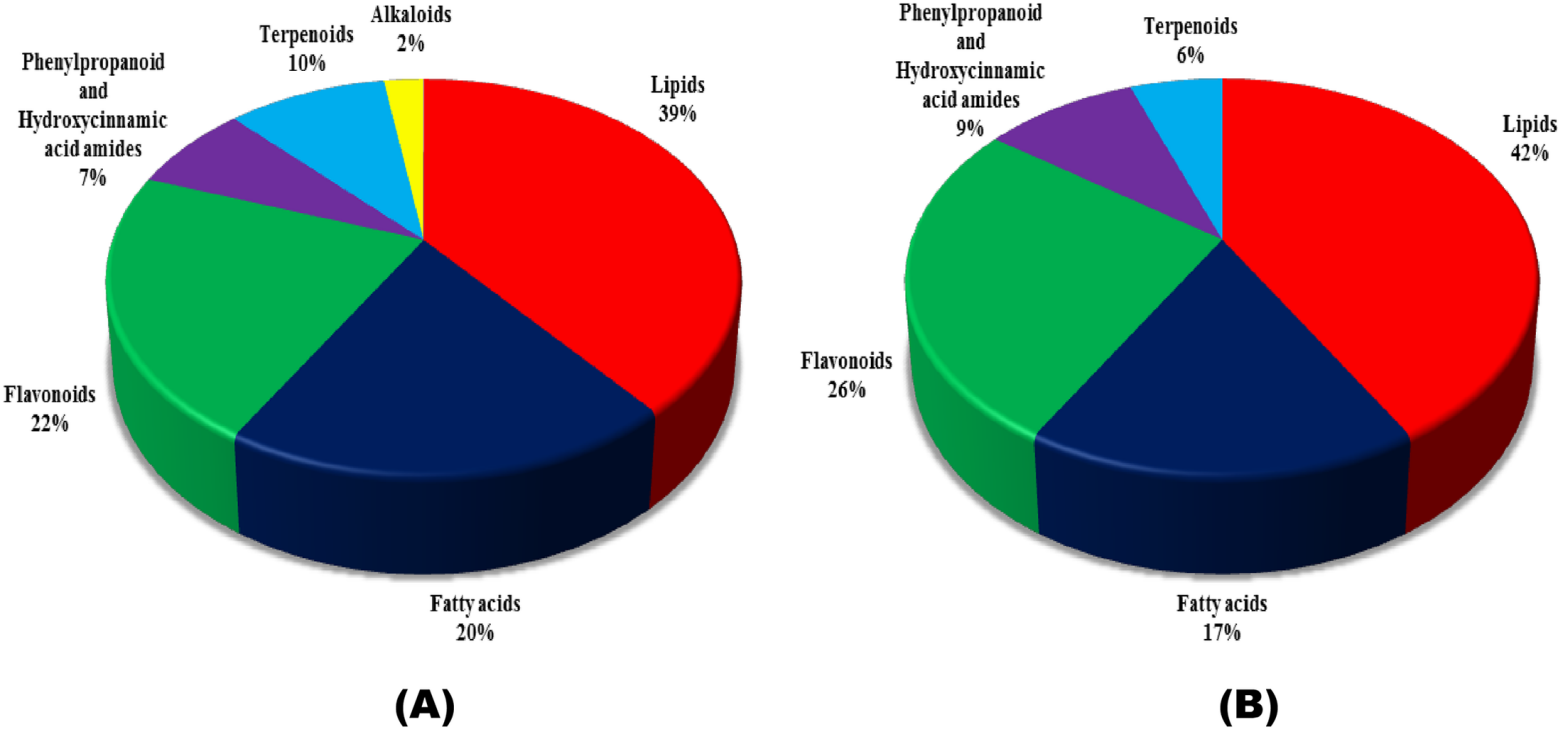
Classification of metabolites detected at 72 hours post *Fusarium graminearum* and water inoculations. Resistant Related Induced (RRI) and Resistant Related Constitutive (RRC) metabolites identified in the study were classified according to their chemical groups. (A) Pie chart shows RRI and (B) shows RRC metabolites classified into various chemical groups.

A total of 546 RRI metabolites were differentially accumulated, of which 41 had relatively high fold change in R-RIL (S1 Table). The RRI metabolites accumulated in higher abundance mainly belonged to phenylpropanoid, flavonoid, lignin, lipid, fatty acid, and terpenoid class of compounds (Table 1; S2 Table; Fig 2A). Metabolites belonging to lipids, in particular, the glycerophospholipids such as, phosphatidic acid (FC = 17.72 & 9.86), phosphatidylcholine (FC = 6.56), phosphatidylinositol (FC = 5.77) were significantly higher in abundance (Table 1). Metabolites belonging to phenylpropanoid biosynthetic pathway, in particular hydroxycinnamic acids (HCAs) such as N-caffeoylputrescine (FC = 5.03) and feruloyl-2-hydroxyputrescine (FC = 3.33) were found to be higher in abundance. Metabolites belonging to flavonoid biosynthetic pathway such as quercetin 3-O-[beta-D-xylosyl-(1->2)-beta-D-glucoside] (FC = 2.59), isovitexin 2''-O-(6‴-feruloyl) glucoside (FC = 2.20), quercitrin (FC = 1.82), 5,7-dimethoxyflavone (FC = 1.64) were few among many that were found in higher abundance. Seven metabolites belonging to fatty acids class of compounds such as 9Z)-(7S, 8S)-Dihydroxyoctadecenoic acid (FC = 6.44), 2,3-Bis (Trimethylsilyl) Oxy-Butanedioic acid Bis (Trimethylsilyl) Ester (FC = 4.10), and cucurbic acid (FC = 2.69). Delcosine (FC = 1.59) an alkaloid was also high in abundance. The compounds identified are known to be involved either in cell wall reinforcement or act as antifungal, antibacterial and antimicrobial compounds, depicting role in FHB resistance.

**Table 1:** High fold change resistance related induced (RRI) and resistance related constitutive (RRC) metabolites identified upon *F*. *graminearum* and mock inoculation of RILs carrying resistant and susceptible alleles of QTL-Fhb2.

Observed Mass (Da)	Exact Mass (Da)	Compound Name	FC	
			RRI	RRC
***Flavonoids***				
596.14	596.14	Quercetin 3-O-[beta-D-xylosyl-(1->2)-beta-D-glucoside]	2.59**	1.47*
770.20	770.21	Isovitexin 2''-O-(6‴-feruloyl)glucoside	2.20**	
448.10	448.10	Quercitrin	1.82**	
282.09	282.09	5,7-Dimethoxyflavone	1.64**	
282.09	282.09	7,4'-Di-O-methyldaidzein	1.64**	
338.12	338.12	Psoralenol	1.58**	
596.15	596.15	Okanin 4'-(6''-p-coumarylglucoside)		2.17*
597.14	597.15	Delphinidin 3-O-beta-D-sambubioside		1.87*
614.16	614.16	Safflomin C		1.80*
596.15	596.15	Okanin 4'-(6''-p-coumarylglucoside)		1.79*
652.16	652.16	Luteolin 7-(6‴-acetylallosyl-(1->2)-glucoside)		1.77*
524.15	524.15	Barbatoflavan		1.69**
***Hydroxycinnamic acid amides and Phenylpropanoids***				
250.13	250.13	N-Caffeoylputrescine	5.03**	
280.14	280.14	Feruloyl-2-hydroxyputrescine	3.31**	
165.08	165.08	L-Phenylalanine	3.14**	
570.20	570.19	Decuroside III		3.74**
330.09	330.10	1-O-Vanilloyl-beta-D-glucose		2.00*
578.20	578.20	Podorhizol beta-D-glucoside		1.94*
386.12	386.12	1-O-Sinapoyl-beta-D-glucose		1.78*
194.06	194.06	Ferulic acid		1.32**
***Terpenoids***				
566.33	566.32	25-Cinnamoyl-vulgaroside	2.58**	
294.19	294.18	Phytuberin	1.81**	
360.16	360.16	Triptolide	1.49**	
520.20	520.19	Brusatol		2.23*
448.23	448.23	Atractyloside A		2.43**
758.44	758.44	Astaxanthin glucoside		1.39*
***Fatty acids***				
314.25	314.25	(9Z)-(7S,8S)-Dihydroxyoctadecenoic acid	6.44*	
438.17	438.17	2,3-Bis(Trimethylsilyl)Oxy-Butanedioic acid Bis (Trimethylsilyl) Ester	4.10*	
212.14	212.14	Cucurbic acid	2.69*	
338.32	338.32	2,4-dimethyl-2-eicosenoic acid	1.62*	
403.31	403.31	N-palmitoyl phenylalanine		2.89**
375.28	375.28	N-arachidonoyl alanine		2.24**
340.33	340.33	Docosanoic acid		1.69*
386.19	386.19	12-hydroxyjasmonic acid 12-O-beta-D-glucoside		1.64**
554.30	554.29	2-O-(beta-D-galactopyranosyl-(1->6)-beta-D-galactopyranosyl) 2S-hydroxytridecanoic acid		1.61*
248.18	248.18	4Z,7Z,10Z,13Z-hexadecatetraenoic acid		1.44**
***Lipids***				
706.46	706.46	PA(15:0/22:6(4Z,7Z,10Z,13Z,16Z,19Z))	17.73*	
682.46	682.46	PA(13:0/22:4(7Z,10Z,13Z,16Z))	9.86*	
739.52	739.52	PC(13:0/20:4(5Z,8Z,11Z,14Z))	6.56*	
834.53	834.53	PI(15:1(9Z)/19:1(9Z))	5.77*	
739.52	739.52	PE(16:0/20:4(5Z,8Z,11Z,14Z))	5.27*	
706.46	706.46	PA(15:0/22:6(4Z,7Z,10Z,13Z,16Z,19Z))		5.81*
686.49	686.49	DG(20:5(5Z,8Z,11Z,14Z,17Z)/22:6(4Z,7Z,10Z,13Z,16Z,19Z)/0:0)[iso]		5.44**
854.50	854.49	PI(16:1(9Z)/20:5(5Z,8Z,11Z,14Z,17Z))		3.30**
***Alkaloids***				
452.26	453.27	Delcosine	1.59**	

Fold change (FC) was calculated based on relative intensity of metabolites: RRC = RM/SM and RRI = (RP/RM)/(SP/SM), where RM = Resistant Mock; SM = Susceptible Mock; RP = Resistant Pathogen; RM = Resistant Mock.

Note:

** significant at *P* < 0.01;

*significant at *P* < 0.05; the significance of RRI was based on RP>RM and SP>SM.

### Resistance related constitutive (RRC) metabolites associated with QTL-Fhb2

A total of 550 RRC metabolites were differentially accumulated, of which 57 were putatively identified (S1 Table). These metabolites belonged to different chemical groups (Table 1; S2 Table; Fig 2B): *Flavonoids (13)*: okanin 4'-(6''-p-coumarylglucoside) (FC = 2.17), delphinidin 3-O-beta-D-sambubioside (FC = 1.87), safflomin C (FC = 1.80); *Phenylpropanoids (5)*: decuroside III (FC = 3.74), 1-O-vanilloyl-beta-D-glucose (FC = 2.0), podorhizol beta-D-glucoside (FC = 1.94), 1-O-sinapoyl-beta-D-glucose (FC = 1.78) and ferulic acid (FC = 1.32); *Fatty acids (9)*: N-palmitoyl phenylalanine (FC = 2.89), N-arachidonoyl alanine (FC = 2.24), and docosanoic acid (FC = 1.69); *Terpenoids (3)*: brusatol (FC = 2.23), atractyloside-A (FC = 2.43), astaxanthin glucoside (FC = 1.39); *Glycerophospholipids (22)*: phosphatidic acid (FC = 5.81), phosphatidylinositol (FC = 3.30), and phosphatidylcholine (FC = 3.26). These high fold change constitutive (RRC) and induced (RRI) metabolites, mainly belonged to phenylpropanoid (hydroxycinnamic acids), flavonoid, glycerophospholipid, and fatty acid classes of compounds, are considered to be responsible for FHB resistance.

### Comparative transcriptome of RILs based on RNA-seq

**Fig 3:**
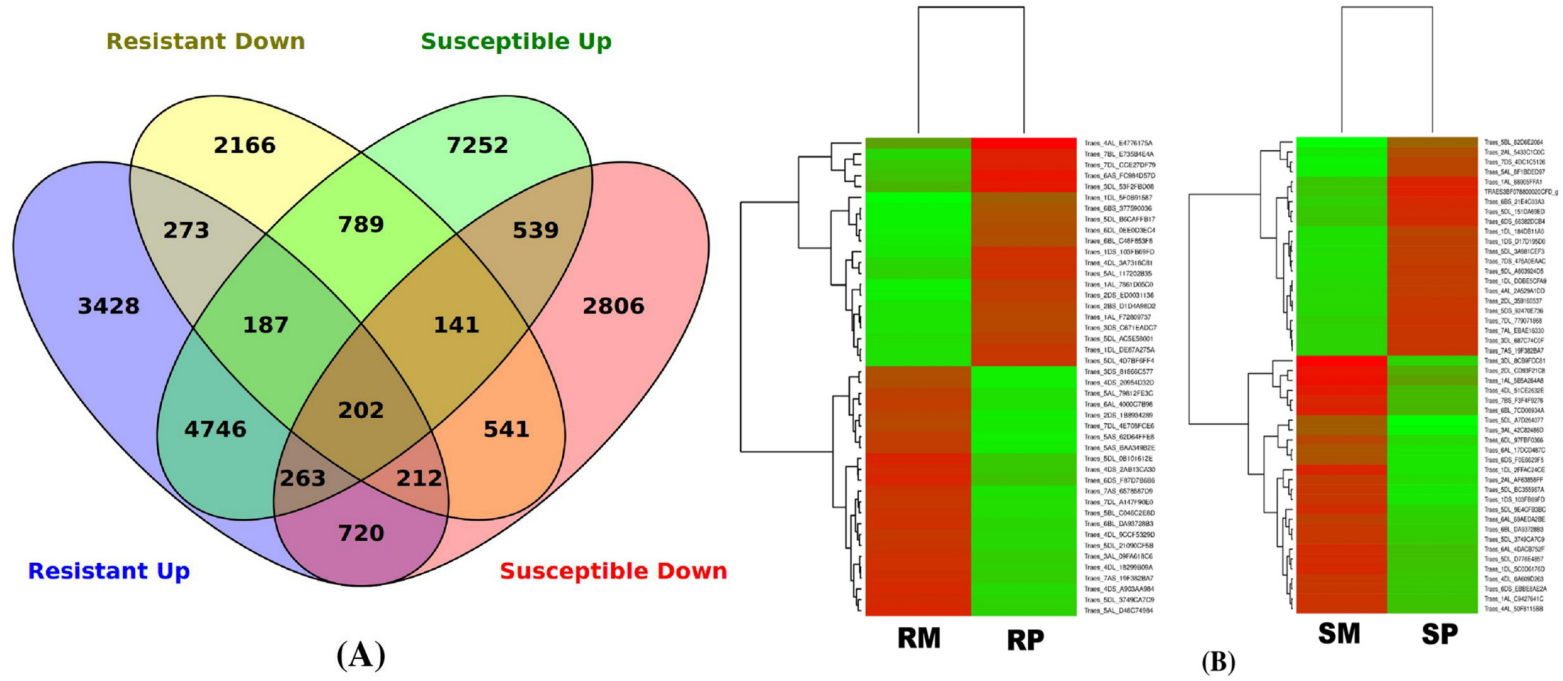
Differentially expressed transcripts in RILs. (A). Venn diagram showing number of transcripts detected as up and down-regulated (*P* < 0.01) in R-RIL and S-RIL, 48 hours post *Fusarium graminearum* inoculation. (B). Heat Maps showing differentially expressed transcripts in R-RIL and S-RIL upon *Fusarium graminearum* and mock inoculations. They show transcripts differentially expressed transcripts between resistant mock (RM) and resistant pathogen (RP) and between susceptible mock (SM) and susceptible pathogen (SP), respectively.

Whole transcriptome analysis (RNA-seq) was done with two genotypes, viz, R-RIL and S-RIL, across four treatments (RP, RM, SP, SM) with three biological replicates for each, collected at 48 hpi. The assembly of sequences, from the 12 sequenced samples, were compared and annotated. Transcriptome analysis using RNA-seq generated 155012 and 165036 transcripts in R-RIL and S- RIL, respectively. The number of transcripts up-regulated and down-regulated in R-RIL and S-RIL are depicted in Fig 3A. The gene ontology analysis classified the transcripts up-regulated in R-RIL and S-RIL based on their involvement in biological processes, molecular functions and cellular component in which they are localized (S1 Fig). The majority of the transcripts depicted their involvement in biological processes such as translation, transcription, response to oxidative stress, lipid metabolism, photosynthesis, DNA repair, protein folding, carbohydrate metabolic processes, trans-membrane transport, suggesting the involvement of various pathways and/or interactions in disease resistance. Differential gene expression (DGEs) analysis classified transcripts as differentially expressed with Log_2_FC values, or transcripts expressed only in either of the genotypes or expressed only upon pathogen inoculation with FPKM values (Table 2; S2 Table). The differentially expressed transcripts (highly up-regulated and highly down-regulated) in R-RIL and S-RIL are shown in the form of heat maps with the respective gene IDs (Fig 3B). Pathway analysis of transcripts showed that majority of transcripts belonged to phenylpropanoid, flavonoid, fatty acid, oxylipin/jasmonic acid, and phospholipid pathways.

**Table 2:** Differentially expressed transcripts in R-RIL and S-RIL upon *Fusarium graminearum* and mock inoculation at 48 hpi. The differential expressions are in log_2_FC values for the resistant genotype and in FPKM values following pathogen inoculation. The up-regulated transcripts were classified according to their biosynthetic pathways. The transcripts localized within QTL-Fhb2 are marked with an asterisk (*).

Gene ID	Annotations	Expression Profile			
		Log_2_FC values (RP/RM)/ (SP/SM)	R-RIL (RP/RM) Log_2_FC values	RP with FPKM values	FPKM fold change (RP/SP)
***Phenylpropanoid and monolignol biosynthesis***					
Traes_5AL_E23B0E6C4	agmatine coumaroyltransferase-2			11.08	
Traes_5DL_7F0CD0F79	caffeic acid 3-o-methyltransferase			10.16	
Traes_3AL_BF387E832	laccase-19			3.76	
Traes_2DL_8BA1CE63D	phenylalanine ammonia-lyase		4.15		
Traes_6BS_CC8E63D7F	4-coumarate: CoA ligase*		1.23		
Traes_7DS_D313DB9ED	laccase				2.26
Traes_6BS_6477278C5	cinnamyl alcohol dehydrogenase*		1.05		
***Flavonoid biosynthesis***					
Traes_6AS_4D2E3A85C	chalcone synthase 8		4.14		
Traes_7DS_3EE1E7974	cinnamoyl reductase		1.47		
Traes_7DL_33BB5BE33	trans-cinnamate 4-monooxygenase	1.30			
Traes_6AL_C82FB1662	dihydroflavonol 4-reductase		3.52		
***Phosphoglycerolipid biosynthesis***					
Traes_1AL_7C3E4A07E	diacylglycerol kinase		1.05		
***Transcription factors***					
Traes_6BS_00E54518B	transcription factor bHLH041*			0.42	
Traes_7DL_5968FA56C	WRKY transcription factor	1.30			
Traes_1AS_36AF74187	R2R3 MYB transcription factor	1.19			
Traes_6AL_BB730675A	MYB-related protein MYB4	1.22			
***Receptor Kinases***					
Traes_4AL_7CC0FB23A	lectin receptor kinase		4.08		
Traes_5AL_6640183AF	serine threonine-protein kinase				2.93
Traes_6DL_BCE522BB4	proline-rich receptor-like protein kinase perk1		2.60		
Traes_5DL_61ECD451B	wall-associated receptor kinase 3		2.45		
***Pathogenesis related proteins***					
Traes_3AL_C932A3F30	peroxidase 2			45.08	
Traes_7DS_F962AB6D6	pathogenesis-related protein 1		4.14		
Traes_7BL_408E23082	chitinase 1	1.41			
Traes_5AS_FAD05211F	pathogenesis-related protein sth-21	1.34			
***Detoxification related transcripts***					
Traes_6BS_4723C124F	abc transporter b family member 4*	1.03			
Traes_2DS_DD9B280C5	udp-glycosyltransferase 85a2		2.65		
Traes_5AS_067CB4CF9	udp-glycosyltransferase 74e1		4.51		
Traes_7BL_D3A25B6C7	pleiotropic drug resistance protein 4 (PDR)		3.61		
Traes_6BS_4EED05084	glutathione s-transferase*			1.95	
***Callose Synthesis***					
Traes_6BS_1668BA98C	callose synthase 7-like*		1.54		

RP = Resistant pathogen, RM = Resistant Mock, SP = Susceptible pathogen, SM = Susceptible Mock. FPKM = Fragment per kilobase of transcript per million mapped reads.

### Genotype-specific transcriptional changes in response to *F*. *graminearum*

The genotype-specific transcriptional modulations are clear indications from the gene ontology classification (S1 Fig), showing the differences in the functional categories of the transcripts in each RIL. The R-RIL dataset, in particular, upon pathogen treatment are considered best to identify the transcripts that would possibly be involved in imparting FHB resistance. Nevertheless, in S-RIL pathogen inoculated dataset, the transcripts down-regulated were not overlooked. The transcripts up-regulated in R-RIL were further classified according to their involvement in various biosynthetic pathways or regulators of gene expression (transcription factors, protein kinases, and secondary messengers) or transcripts belonging to RR-proteins involved in DON detoxification (Table 2). The transcription factor *bHLH041* (FPKM = 0.42) was detected only in RP, suggesting its role in FHB defense. Apart from *bHLH*, transcription regulatory genes like *WRKY*, *R2R3 MYB* and *MYB-4* were up-regulated in R-RIL (Table 2). The transcripts belonging to phenylpropanoid pathway genes such as agmatine coumaroyltransferase-2 (*ACT*, FPKM = 11.08), caffeic acid 3-o-methyltransferase (*CoMT*, FPKM = 10.16), laccase (FPKM = 3.19) were detected only in RP, while phenylalanine ammonia-lyase (*PAL*, FC = 4.15) and 4-coumarate: CoA ligase (*4CL*, FC = 1.23) were detected only in R-RIL. Cinnamyl alcohol dehydrogenase (*CAD*) was detected in both the RP and SP, with higher expression in RP. Chalcone synthase 8 (*CS8*), cinnamoyl reductase (*CR*), and dihydroflavonol 4-reductase (*DHFR*) genes of flavonoid biosynthetic pathway were up-regulated in RP. Receptors kinases like lectin receptor kinase (*LRK*, FC = 4.08), proline-rich receptor-like protein kinase perk (FC = 2.60), wall-associated receptor kinase 3 (*WAK3*, FC = 2.45) were also up-regulated in RP. Transcripts belonging to PR protein, peroxidase 2 (*PR2*, FPKM = 45.08) was detected only in RP. Several other PR proteins such as *PR1*, *PR2*, and chitinases were also up-regulated in RP. Transcripts involved in the detoxification were highly up-regulated in RP such as, UDP-glycosyltransferases, multidrug resistance proteins, pleiotropic drug resistance proteins, ABC transporters and glutathione S- transferases (Table 2; S2 Table). All the transcripts up-regulated in resistant genotype were reported to be involved in FHB resistance, thus implicating the involvement of a hierarchy of genes and/or interactions in imparting FHB resistance in wheat.

### Genetic controls underlying QTL-Fhb2

**Fig 4:**
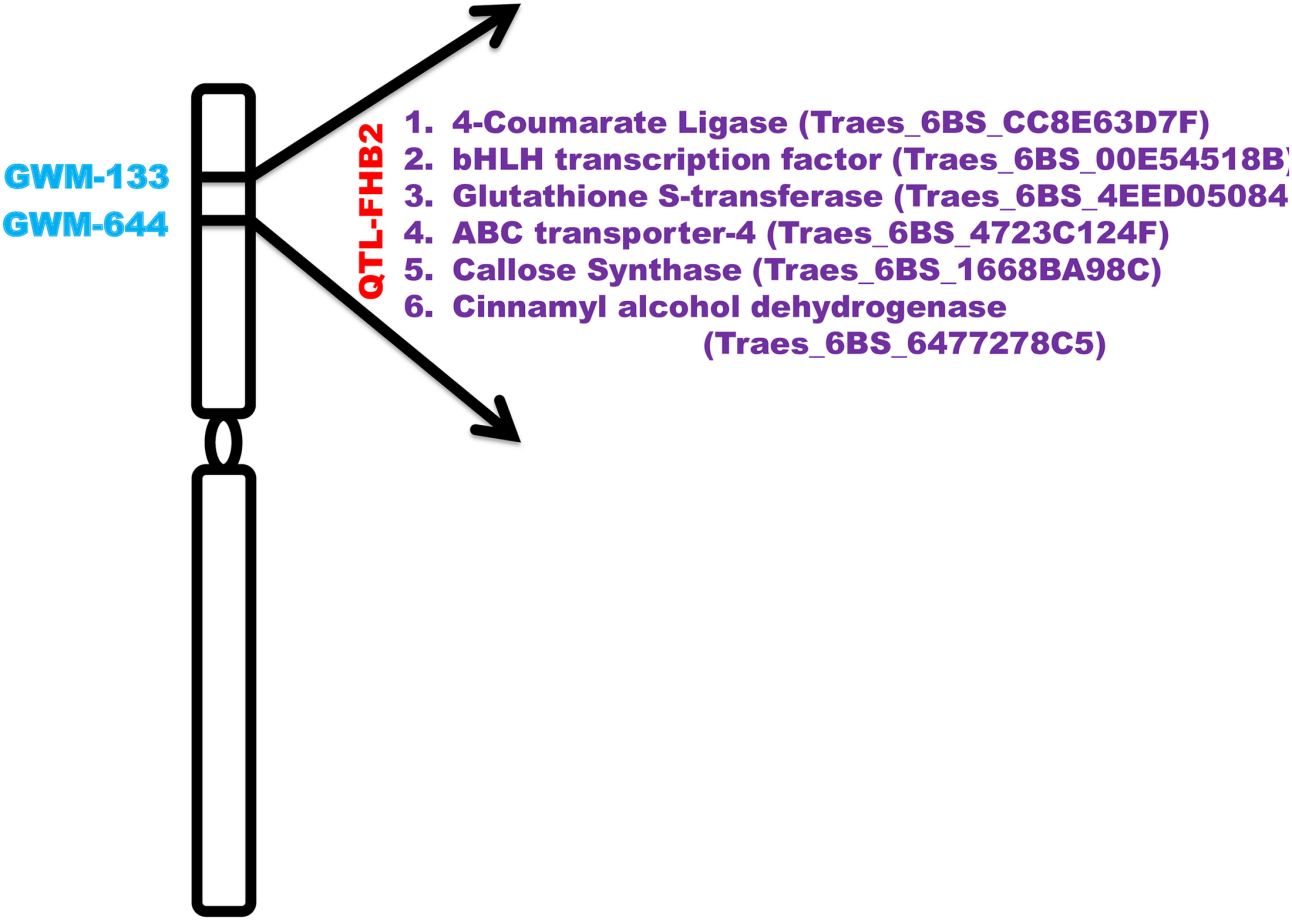
Wheat chromosome 6B, depicting the physical location of QTL-Fhb2 (marked red), on short arm of the chromosome 6B, flanked by two SSR markers GWM-133 and GWM-644 (marked blue). The genes localized within the QTL locus were identified using metabolo-transcriptomics approach, which are shown within two arrow marks, marked purple in text. The corresponding gene Ids are given in parenthesis.

The markers flanking the QTL were sequenced and the region (sequence) within the two flanking markers was considered as QTL-Fhb2 region using wheat survey sequence available (http://wheat-urgi.versailles.inra.fr/). The transcripts aligning to the 6BS reference genome were pulled out separately and furthermore, the transcripts aligning to the sequence within the flanking marker co-ordinates were considered as genes localized within QTL-Fhb2 region. Based on high FC metabolites and transcripts in R-RIL we were able to localize six putative candidate genes within the QTL-Fhb2 region that were associated with biotic stress resistance functions (Fig 4). The putative candidate genes localized within the region were: *4CL*, *bHLH041* TF, *GST*, *ABC-4*, *CS*, and *CAD*. The expression values for these genes localized within the QTL-Fhb2 region are presented in Table 2 (transcripts marked with asterisk (*)). The list of all the genes localized within the QTL-Fhb2 region is provided in S3 Table.

### Confirmation of gene expression based on qRT-PCR

**Fig 5:**
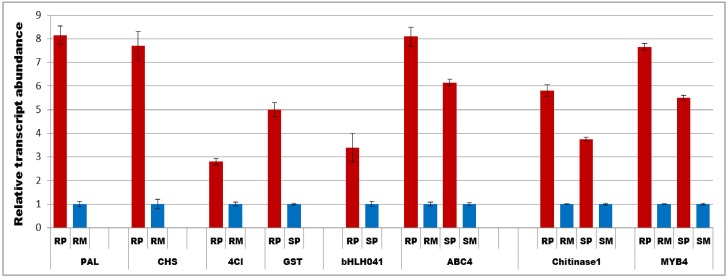
Quantitative Real time PCR (qRT-PCR) showing transcript abundances of selected genes at 48 h post *F*. *graminearum* and water inoculation. The relative transcript abundance were calculated compared to mock treatments and for transcripts which were only expressed in pathogen treated treatments, susceptible pathogen was used to compare the abundance in resistant pathogen. RP = Resistant pathogen, RM = Resistant mock, SP = Susceptible pathogen and SM = Susceptible mock. PAL-Phenylammonia lyase, CHS = chalcone synthase, 4Cl = 4 coumarate CoA-ligase, GST = Glutathione S-transferase, bHLH041 = basic helix loop helix transcription factor, ABC4 = ABC transporter4.

To validate the RNA-seq data a qRT-PCR analysis was carried out for a few selected genes, such as, *4CL*, *bHLH041*, *ABC-4*, *GST*, *chitinase1*, *CHS*, *PAL* and *MYB4*. The expression values obtained from qRT-PCR analysis for the selected genes were similar to RNA-seq confirming the reproducibility of transcriptome data (Fig 5).

## Discussion

Resistance in wheat against FHB is quantitative and more than 100 QTLs with major or minor effects have been identified and mapped on all wheat chromosomes, expect on 7D. However, the genetic controls underlying these resistant QTLs are yet to be revealed. These QTL regions, even when they are fine mapped, contain several genes, including those conferring resistance. Identification of candidate genes and their resistance functions are crucial to map the hierarchical network of genes involved in the biosynthesis of a given or set of RR metabolite(s), which directly suppresses the pathogen progress. The *R* genes biosynthesizing these RR metabolites are regulated by other *R* genes; thus the functional hierarchy must be confirmed before an individual gene can be transferred to a susceptible genotype [12]. Also these specific candidate genes can be transferred without linkage drag effects based on genome editing tools. In view of this, an attempt was made to dissect one of the major FHB resistant QTL*-*Fhb2 to identify the underlying genetic controls. Integrating two systems biology disciplines, metabolomics and transcriptomics, we were able to identify putative genes localized within the QTL-Fhb2 region and their plausible mechanisms of resistance.

### Metabolic profiling identified potential FHB resistance biomarkers

Non-targeted metabolic profiling of genotypes with contrasting levels of FHB resistance identified several RR metabolites, highly accumulated, in different metabolic pathways such as glycerophospholipid, phenylpropanoid (HCAAs), flavonoid, fatty acid and terpenoid. Glycerophospholipids like phosphatidic acid (PA) (FC = 17.72), phosphatidylcholine (FC = 6.56), phosphatidylinositol (FC = 5.77) are known to be deposited to enforce cell walls. Furthermore, these are also known to perceive and transmit signals activating downstream genes eventually regulating *R* genes to biosynthesize RR metabolites and RR proteins [43]. These compounds are either converted into bioactive lipids (components of lipid bilayer of cell membrane) or stay as soluble molecules (messengers), further binding to the downstream mitogen activated protein kinases (MAPK), further affecting the enzymatic activities, vesicle trafficking and ion fluxes [43]. PA acid is an important secondary messenger in plant stress signaling [44]. These stresses involve pathogen attack, salinity, cold, drought, heat and wounding. In regard to pathogen response, PA acid has been shown to accumulate in response to various elicitors such as xylanase, flagellin, and chitosan [45]. Interestingly, a gene diacylglycerol kinase (DGK) that catalyzes the conversion of structural lipids (PC, PE, PS) into PA was upregulated in our study. This clearly illustrates that the early membrane modifications and their involvement in further activating defense responses are very crucial.

Rachis resistance in wheat is mainly due to the deposition of HCCAs [6]. In our study, we detected a higher abundance of two HCCAs in particular, N-Caffeoylputrescine (FC = 5.03) and Feruloyl-2-hydroxyputrescine (FC = 3.31) upon *F*. *graminearum* inoculation. Suberin, a complex, intractable biopolymer, with polyaromatic domain of HCCAs, is deposited apoplastically between the primary cell wall and plasma membrane to enforce cell walls [46]. Once, the cell walls are thickened the pathogen won’t be able to spread within the spike, through the rachis, thus imparting high levels of rachis resistance. Resistance in NIL carrying resistant alleles of QTL-Fhb1 was reported to be due to the deposition coumaroylputrescine, feruloylputrescine, coumaroylagmatine, cinnamoylserotonin, feruloylagmatine, p-coumaroylserotonin [6]. HCCAs such as feruloylputrescine, p-coumaroyltyramine, N-feruloyltyramine, 4-coumaroyl-3-hydroxyagmatine, feruloylagmatine, 4- coumaroylagmatine, terrestriamide, and feruloylserotonin were reported to impart late blight resistance in potato [30,33]. HCCAs imparted resistance in tomato against *Pseudomonas syringae* [47], in maize against *F*. *graminearum* [48], in onion against *Botrytis allii* [49]. These HCCAs are not only involved in cell wall thickening, but also they possess antioxidant, antiviral, antibacterial and antifungal activities [47]. Silencing of *St-WRKY1* reduced not only HCAAs, but also increased susceptibility of potato to late blight, thus confirming the role of HCAAs in resistance. These HCCAs can be used as markers to screen FHB resistance genotypes, because RR metabolites being the end products of *R* gene function, guarantee resistance [14].

Apart from HCCAs, we also have identified several flavonoids with high abundance, as RRI and RRC metabolites, such as, quercetin 3-O-[beta-D-xylosyl-(1->2)-beta-D-glucoside], isovitexin 2''-O-(6‴-feruloyl) glucoside, quercitrin, 5,7-dimethoxyflavone, 7,4'-di-O-methyldaidzein, psoralenol, fisetinidol-4beta-ol 3,4,7,3',4'-pentamethyl ether, glyceollin I, and 3,7-di-O-methylquercetin. The growth of *Fusarium* and macroconidia formation is completely inhibited by dihydroxyquercetin [50], and of the fungus *Neurospora crassa* by quercetin 3-methyl ether and its conjugated glucosides [51]. The deposition of flavonoid conjugates (of glucoside and methoxy) was higher in rachis tissues of NIL carrying resistance alleles of genes in QTL-Fhb1 [6]. The involvement of both preformed and induced flavonoids in plant defense against pathogens, herbivores, and environmental stress is well documented [52]. In resistant barley several flavonoids were accumulated in high abundance upon *F*. *graminearum* inoculation [35,38].

We detected high fold accumulation of preformed and induced free fatty acids such as dihydroxyoctadecenoic acid, 2,3-bis(trimethylsilyl)oxy-butanedioic acid bis (trimethylsilyl) ester, cucurbic acid, 2,4-dimethyl-2-eicosenoic acid, N-palmitoyl phenylalanine, and N-arachidonoyl alanine. Fatty acids are not only part of structural constituents, but also act as secondary messengers and regulators of signal transducing molecules or transcription factors [53]. Arachidonic acid acts as an elicitor in plant defense response to phytopathogens [54]. Several free fatty acids were accumulated in barley upon *F*. *graminearum* invasion [38]. The antifungal capabilities of octadecenoic, tetradecanoic, docosanoic, butenoic acid have been reported [55]. Hence, these fatty acids may be crucial components of FHB resistance in wheat, not only acting as physical barriers, but also as antimicrobials.

### Transcriptome changes provided key insights to genetic reprogramming upon pathogen invasion

Transcripts belonging to phenylpropanoid and flavonoid pathways, including receptor kinases, transcription factors, detoxification, and signaling genes were highly regulated, following pathogen inoculation (Table 2). The elicitors produced by pathogen are perceived by plant membrane receptors. In our study, we found higher transcript abundances of lectin receptor kinase (*LRK*), serine threonine-protein kinase (*STPK*), proline-rich receptor-like protein kinase (PERK1), and wall-associated receptor kinase 3 (*WAK*3). The role of LRKs [56], *STPKs* [57], and *WAKs* [58] in plant defense is well documented. The overexpression of *WAK1* in *Arabidopsis thaliana* conferred higher resistance to *Botrytis cinerea* [58]. The resistance to fungal pathogen *Magnaporthe grisea* was due to a G-type lectin receptor kinase (*Pi-d2)* in rice [59]. These receptor kinases transduce signals downstream, activating several groups of TFs. The TFs belonging to *bHLH*, *WRKY* and *MYB* groups were up-regulated, depicting their involvement in regulating downstream *R* genes that biosynthesize RR metabolites and proteins. In our study, the phenylpropanoid pathway genes, such as, agmatine coumaroyltransferase (*ACT*), caffeic acid 3-o-methyltransferase (*CoMT*), laccase, phenylalanine ammonia-lyase (*PAL*), 4-coumarate CoA ligase (*4CL*), and cinnamyl alcohol dehydrogenase (*CAD*) were highly expressed in R-RIL. *PAL*, a hub gene, that biosynthesizes precursor for phenylpropanoid biosynthetic pathway, was highly up-regulated in Sumai-3 upon *F*. *graminearum* invasion [41]. *ACT* which is localized within wheat FHB resistant QTL-2DL imparts high rachis resistance by cell wall thickening [60]. *4CL* is an important *R* gene for both lignin and flavonoid biosynthesis, and was induced in cucumber against powdery mildew [61], cotton against wilt fungus *Verticillium dahlia* [62], and potato against *Phytophthora infestans* [30]. Laccase and peroxidase (*POD*) involved in lignin biosynthesis were up-regulated in our study, emphasizing an increased lignification of cell walls around infection site in R-RIL. In our study, the *peroxidase* was highly expressed in RP (FPKM = 45.08). The involvement of *POD* in the defense responses of wheat to *Fg* infection has been reported [39]. Genes involved in flavonoid biosynthesis like chalcone synthase8 (*CHS8*), cinnamoyl reductase (*CR*) and dihydroflavonol 4-reductase (*DHFR*) were detected only in R-RIL. The disruption of flavonoid pathway significantly reduced flavonoid metabolites [61]. Resistance in wheat to the hemibiotrophic pathogen, *Septoria tritici* was due to a higher accumulation of *CHS* transcripts [63]. RR proteins restrict the spread of pathogens. In our study, we detected several PR proteins such as *chitinase*, *peroxidase*, and *PR1* with higher expressions. Chitinases are very important in plant defense against many fungal pathogens, as they degrade the fungal cell walls, which are primarily made up of chitin. Expression of barley class-II chitinase gene in wheat conferred high level of resistance against *F*. *graminearum* under greenhouse and field conditions [64]. Expression of rice chitinase enhanced resistance against *Magnaporthe grisea* in rice [65], *Uncinula necator* in Italian ryegrass [66], and *Puccinia coronata* in grapevine [67].

Mycotoxins produced by *Fg*, such as trichothecenes, play a major role in pathogenesis, especially DON, a well-known virulence factor. Therefore, the resistance to DON is crucial to confer enhanced FHB resistance [68]. Mutant *Fg* strains (unable to produce DON) showed reduced FHB severity [69]. In previous studies, it has been reported that DON is converted into less toxic DON-3-O-glucoside (D3G) [70,71]. However, several genes are involved in DON reduction in plants, such as multidrug-resistant protein, multidrug resistance-associated protein, UDP-glycosyltransferase and ABC transporters were detected with higher transcript abundances upon *Fg* inoculation in wheat cv. Nobeokabouzu-komugi [17]. The accumulation of glutathione S-transferase (*TaGSTF5*) in wheat resistant cv. Ning7840 upon *Fg* invasion was significantly higher [72]. Similarly, in our study we found higher expressions of ABC transporter b family member 4, UDP-glycosyltransferase 85a2, UDP-glycosyltransferase 74e1, pleiotropic drug resistance protein 4 and glutathione S-transferase in R-RIL upon pathogen inoculation. Transgenic Arabidopsis and wheat expressing a barley *UDP-glucosyltransferase* (*HvUGT13248*) detoxifies deoxynivalenol and provides high levels of resistance to *F*. *graminearum* [68,73]. ABC transporter proteins (yeast *PDR5* like) confined to plasma membrane confers partial resistance against trichothecenes in wheat by serving as drug efflux pumps [74]. The higher transcript abundances of detoxification genes, clearly explain the reduced levels of DON accumulation in R-RIL, thus contributing to FHB resistance.

### QTL-Fhb2 imparts resistance through additive effects of cell wall reinforcement and DON detoxification

**Fig 6:**
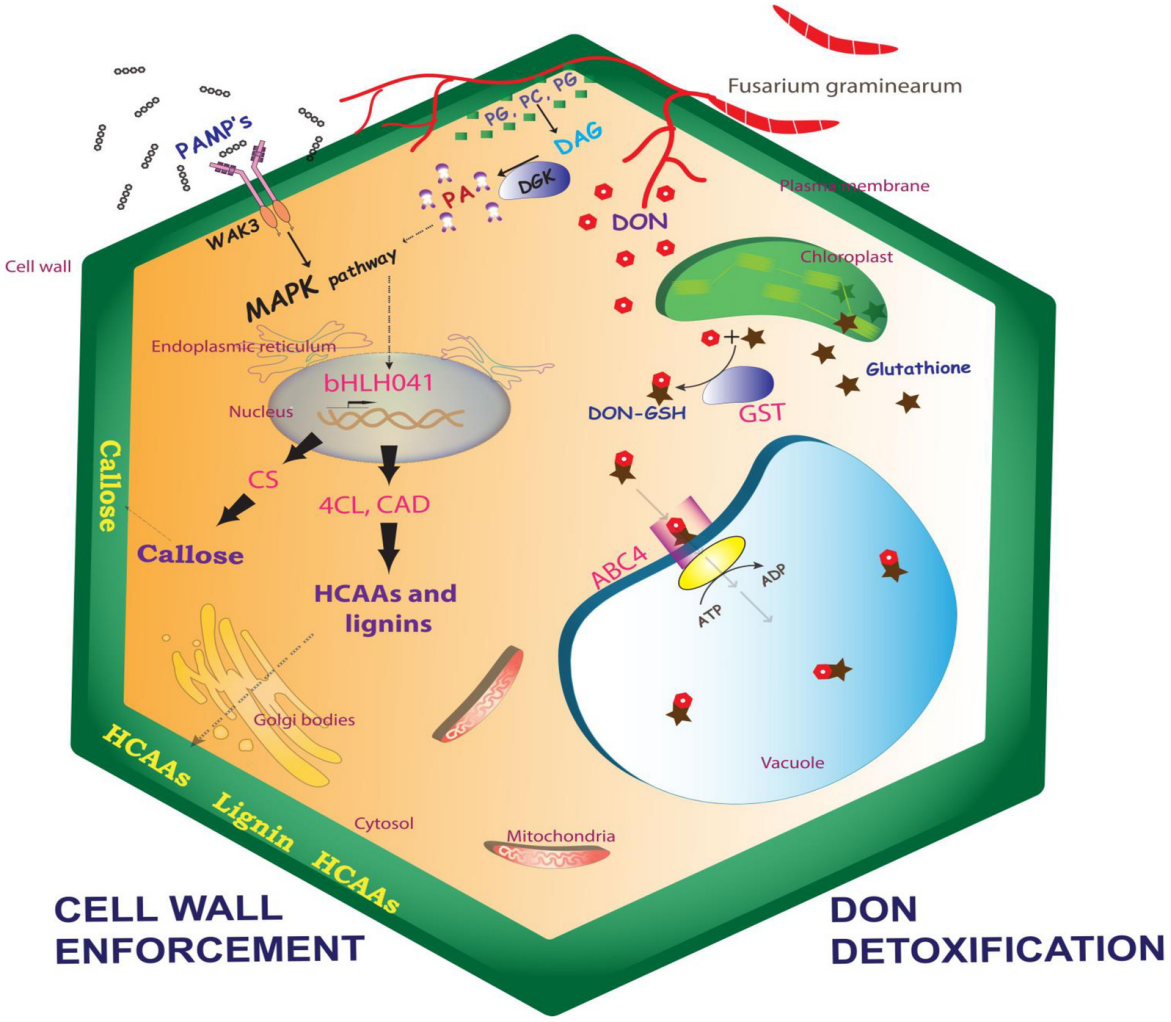
Hypothetical model for FHB resistance in wheat line carrying resistant alleles of R genes in QTL-Fhb2. Based on our findings we propose that the QTL-Fhb2 imparts high rachis resistance through combined effects of cell wall reinforcement and DON detoxification.

The markers flanking the QTL locus were sequenced and the sequence (http://wheat-urgi.versailles.inra.fr/) within the two flanking markers was retrieved and the potential *R* genes in that region were identified (Fig 4). QTL-Fhb2 was consistently mapped on chromosome 6BS, conferring high rachis resistance [23,24]. Based on high FC metabolites and transcripts, we identified *4CL*, *CS*, *bHLH041*, *GST*, *ABC4*, and *CAD* as putative candidate *R* genes localized within the QTL-Fhb2 region. Based on our study, we propose a hypothetical model for FHB resistance in wheat line carrying resistant alleles of genes in QTL-Fhb2 (Fig 6). The importance of each candidate gene in the model on plant defense is discussed.

### 4CL, CS and CAD confer resistance through cell wall reinforcement

#### 4-Coumarate CoA: Ligase (4CL)

We detected a higher transcript abundance (FC = 1.23) of *4CL* in R-RIL. 4CL is an important enzyme that catalyzes the conversion of coumaric, ferulic, caffeic, and sinapic acids into hydroxycinnamoyl-CoA thiol esters, which further serve as precursors in lignin, flavonoid, polyphenol, coumarin and suberin biosynthesis [75]. Two HCCAs, N-Caffeoylputrescine (FC = 5.03) and feruloyl-2-hydroxyputrescine (FC = 3.31) were in high abundance, showing a higher expression of *4CL* to biosynthesize them in R-RIL than in S-NIL. Increased expression of *4CL* increased HCCAs accumulation in potato against *P*. *infestans*, imparting resistance through thickening of cell walls [28–30,33]. Interestingly, we also detected higher abundances of metabolites and transcripts from lignin and flavonoid pathways, implicating the role of *4CL* in the biosynthesis of precursors. Silencing of *4CL* reduced lignin content in switch grass [76], and poplar [77]. As *4CL* is a hub *R* gene in the phenylpropanoid pathway leading to the biosynthesis of lignins and flavonoids, we consider this to be a potential candidate *R* gene conferring high rachis resistance to FHB.

#### Callose synthase (CS)

Plants restrict the spread of pathogens through deposition of an RR metabolite, such as callose (β-1,3-glucan) to form cell wall appositions or papillae [78]. Papillae are complex structures formed around the invading hyphae, which are produced in-between plasma membrane and the cell wall. The papilla is composed of different classes of compounds such as, phenolics, reactive oxygen species, cell wall proteins and glucans [79]. In our study, we also detected a higher transcript abundance of *callose synthase*. Callose synthase 5 (*CalS5*) in *Arabidopsis thaliana* plays a predominant role in the synthesis of the callose wall and callose plugs, and containment of powdery mildew hyphae in Arabidopsis [80]. *Arabidopsis thaliana* callose synthase *PMR4* expression in barley increased penetration resistance to powdery mildew [81]. Hence, we consider callose synthase as one of the integral candidates in FHB defense.

#### Cinnamyl alcohol dehydrogenase (CAD)

*CAD* is a key enzyme in lignin biosynthesis that catalyzes reduction of cinnamaldehydes into cinnamyl alcohols, the last step of monolignol biosynthesis, before oxidative polymerization in the cell wall [82]. Lignin is a complex phenolic polymer which is deposited in the cell walls of many plants. Deposition of lignin (lignification) is known to confer resistance against invading pathogens. Lignification thickens the cell wall and makes it difficult for fungal appressoria to penetrate into the cell [83]. It also makes cell walls more water resistant, and in turn, less accessible to cell wall degrading enzymes [83]. Gene expression profiling and silencing showed monolignol biosynthesis is very important in penetration defense in wheat against powdery mildew invasion [84]. Cinnamyl alcohol dehydrogenase-C and D play a crucial role conferring resistance in Arabidopsis against bacterial pathogen *Pseudomonas syringae* pv. tomato [85]. *AtCAD1* is involved in lignification of elongating stems in *Arabidopsis thaliana* [86]. *CAD* was upregulated in NILs containing QTL-Fhb1, upon pathogen invasion [6].

### ABC transporter and GST aiding resistance through DON reduction

#### ABC transporter-4 (ABC-4)

DON inhibits eukaryotic protein synthesis and increases the virulence of *F*. *graminearum* by suppressing RR protein and metabolite biosynthesis in plants. A wheat ABC transporter (*TaABCC3*.*1*) imparts DON tolerance [87]. A *TaABCC* (ABC transporter C family) gene within FHB resistant QTL-2DS conferred resistance by reducing DON accumulation [88]. *TaABCC* gene underlying wheat resistance QTL-Fhb1 imparts FHB resistance [89]. Similarly, in our study we identified higher transcript abundance of ABC transporter b-family member 4 and consider this to play a significant role in rachis resistance, by reducing DON for virulence.

#### Glutathione S-transferase (GST)

GSTs play an important role in plant resistance against biotic and abiotic stresses [90]. These are dimeric enzymes that catalyze the conjugation of electrophilic molecules to glutathione (GSH) [91]. DON the *Fusarium* virulence factor upon conjugation with GST is presumably detoxified, thus decreasing the pathogenicity of the fungus [27,92]. GSTs from barley are reported to detoxify DON [70]. Glutathione S-transferase genes were induced in *Nicotiana benthamiana* upon *Colletotrichum destructivum* and *C*. *orbiculare* infection and a GST gene was implicated in resistance [91]. GSTs were induced in potato upon *Phytophthora infestans*, wheat upon *Erysiphe graminis* and Arabidopsis upon *Peronospora parasitica* infection [93]. The expression of GST gene from *Lilium regale* conferred resistance to *Fusarium oxysporum* in tobacco [94]. Similarly, we presume that the GST identified in our study, reduces the virulence of pathogen by detoxification of DON, and in turn, aid in enhancing FHB resistance.

#### *bHLH041*: A novel candidate for FHB resistance

The bHLH group of transcription factors shares a basic helix loop helix protein structure. The size of these transcription regulators varies anywhere from 60–100 amino acids and consists of two highly conserved domains [95]. The N-terminal basic domain functions as a DNA-binding domain, and the second basic domain which is separated by a loop, determines the dimerization capacity of a protein [95,96]. These transcription regulators usually bind to a consensus sequence known an E-box (CANNTG) [97]. The role of bHLH transcription regulators in plant response to pathogen attack has been well documented [96,98]. A transcription regulator *TabHLH060* was highly expressed in wheat leaves upon invasion by an obligate pathogen *Puccinia striiformis* f. sp. *tritici* [96]. In our study, the transcription regulator *TabHLH041* was detected only in RP (FPKM = 0.42). Further functional characterization and the identification of its potential downstream targets should increase our understanding about its role in FHB defense reactions.

Our study identified several important *R* genes localized in the QTL-Fhb2. Even though this QTL was fine mapped it contains several genes with different mechanisms of resistance, but acting cumulatively to impart high level of rachis resistance. These genes should be sequenced to verify if they are functional in R-RIL, but nonfunctional in S-RIL. These polymorphic candidate genes identified here can be used in breeding, following validation of gene resistance functions based on silencing these genes in resistant genotype. The functional alleles of these genes can be used to replace the alleles in susceptible commercial cultivars, if nonfunctional, based on genome editing.

## Conclusion

FHB resistance is polygenic in nature; many *R* genes with major and/or minor effects contribute cumulatively conferring resistance. The mapped FHB resistant QTLs localize several genes governing resistance. We dissected FHB resistant QTL-Fhb2, based on integrated metabolo-transcriptomics approach and putatively identified six resistance genes. These genes confer structural resistance through cell wall reinforcement, reducing the spread of pathogen through rachis within the spike. Several other genes detoxify DON, the virulence factor, and thus reducing the severity of the disease. In conclusion, we report that the wheat resistant QTL-Fhb2 confers high rachis resistance through combined effects of cell wall enforcement and reduction of DON. The candidate genes identified here, upon functional characterization, can pave the way to develop highly FHB resistant genotypes.

## Materials and Methods

### Genetic background of RILs carrying contrasting alleles of QTL-Fhb2

The recombinant inbred population of 1,440 F_2:7_ lines were developed using single seed descent method, through a cross between BW-278 (AC Domain*2/Sumai-3 = FHB resistant parent) and AC Foremost (HY320*5/BW553//HY320*6/7424-BW5B4 = FHB susceptible parent) [23]. The resistant parent BW-278 is a descendant of Sumai-3, a Chinese wheat cultivar that exhibits high rachis resistance and is the source of major FHB resistance QTLs. However, BW-278 is known to lack QTL-Fhb1 resistance alleles on chromosome 3BS. The RILs derived segregated for three known FHB resistance QTLs on chromosomes 3BSc, 5A (QTL-Fhb5), and 6B (QTL-Fhb2). The lines were further genotyped using simple sequence repeat (SSR) markers on chromosome 6B (WMC104, WMC397, GWM219), 5A (GWM154, GWM304, WMC415), and 3BS (WMC78, GWM566, WMC527) to select RILs homozygous susceptible for QTL intervals on 3BSc, 5A, and recombinant for the interval on 6B carrying the FHB resistance gene [23]. The seeds of FHB resistant and susceptible RILs carrying contrasting alleles of QTL-Fhb2 were obtained from Dr. Curt McCartney (AAFC, Winnipeg, MA). The highly resistant RIL = PbI-170 (carrying resistance alleles of QTL-Fhb2, R-RIL) and the highly susceptible RIL = QeJ-004 (carrying susceptible alleles of Fhb2, S-RIL) were selected for this study taking into consideration the phenotypic data, both greenhouse and field, provided by Dr. McCartney.

### Plant and pathogen production, and inoculation

All experiments were conducted in greenhouse as a randomized complete block design, with two RILs (R-RIL and S-RIL), two inoculations (pathogen and mock as control) and three to five replications over time, depending upon the nature of the experiment. In each pot, 4 seeds were sown at 5 d intervals. Plants were grown at 25 ± 3°C, 70 ± 10% relative humidity and 16 hours of photoperiod throughout the growing period. Plants were irrigated every day and fertilized at 15 d intervals with 20-20-20 NPK, and the trace elements according to the growth stage of the plants [6]. The *F*. *graminearum*, isolate Z-3639 was initially cultured on potato dextrose agar (PDA, DIFCO Laboratories Detroit, Michigan, USA) medium to produce mycelia and then in rye-B agar medium with incubation under UV light for the production of macroconidia [99]. Cultures grown for a week were used to prepare macroconidial suspension for inoculations. The final concentration of spore in the suspension was adjusted to 10^5^ ml^-1^ using sterilized distilled water and 10 μl suspension was inoculated per spikelet [20]. Plants were inoculated at about 50% anthesis (GS = 60–65) and were covered with plastic bags for 48 hours to maintain high humidity in order to facilitate initial infection.

### Phenotyping of RILs

Disease severity analysis and fungal biomass quantification were carried out to phenotype RILs, for FHB resistance. For disease severity analysis, a single alternate pair of spikelets, in the middle of a spike of each RIL was inoculated with *F*. *graminearum* macroconidial suspension. The total number of spikes inoculated per replication were ten and the total number of replications were five. The number of spikelets diseased were recorded every three days until 21 days post inoculation (dpi) and the proportion of spikelets diseased (PSD) and the area under disease progress curve (AUDPC) was calculated. For fungal biomass quantification, three alternate pairs of spikelets per spike per RIL were inoculated with *Fg* spore suspension and distilled water, separately. The number of spikes inoculated per replication and the number of total replications were three. 7dpi, six successive pairs of spikelets (three alternate pairs inoculated and three uninoculated) were harvested; the rachis region underlying these six spikelets was collected. The total DNA was extracted from rachis tissues using DNeasy Plant Mini Kit (Qiagen, Germany). The concentration of DNA was normalized to 20 ng/μl and the fungal biomass was quantified using *F*. *graminearum* specific *Tri6* as target gene and wheat specific *Actin* as a housekeeping gene [20]. The relative gene copy number of *Tri6* was calculated following 2^−ΔΔC^_T_ method [100].

### Sample collection, metabolite extraction, metabolic profiling, and data analysis

For metabolic profiling, the experiment was conducted in a randomized complete block, with two genotypes (R-RIL and S-RIL), two inoculations (pathogen = P and mock = M) and five replications per treatment. Three alternate pairs of spikelets of a spike were inoculated with *F*. *graminearum* spore suspension (P) or distilled water (M). 7dpi, six successive pairs of spikelets (three alternate pairs inoculated and three uninoculated) were harvested and the rachis region underlying these six spikelets was separately collected, ground in liquid nitrogen using pre-chilled mortar and pestle. 100 mg of tissue samples were weighed in 2 ml sterilized micro-centrifuge tubes and used for metabolite extraction. Metabolites were extracted using absolute methanol followed by 60% methanol in order to extract polar, semi-polar and non-polar metabolites [101]. The metabolites were analyzed using LC-ESI-LTQ-Orbitrap-MS. Randomization of samples was done to avoid any structural errors associated with the liquid chromatography and high resolution mass spectrometry (LC-HRMS = LC-LTQ-Orbitrap) analysis. The output data files obtained from LC-MS analysis were first converted into mzXML/.cdf and were exported to MZmine2 software for peak deconvolution, peak detection, spectral filtering and normalization of peaks [102].

The abundance of peaks were subjected to paired *t*-test (comparison of two treatments at a time) to identify treatment significant metabolites. Treatment significant metabolites with *P* < 0.05 were retained for further analysis. Metabolites with higher abundance in resistant genotype (R-RIL) than in susceptible genotype (S-RIL) were considered as resistance related (RR) metabolites. A RR metabolite based on inoculation (M) was considered as constitutive resistance related constitutive (RRC = RM>SM) metabolite. A metabolite with significantly higher abundance in the pathogen inoculated treatments than in mock inoculated treatments was considered as a pathogenesis related (PR) metabolite, in resistant (PRr = RP>RM) or susceptible (PRs = SP>SM) genotypes. A PRr metabolite in a resistant genotype with abundance greater than that in susceptible pathogen (PRs) inoculated was considered as a resistance related induced metabolite (RRI = (RP>RM) > (SP>SM)) metabolite [6,10]. The resistance metabolites were identified with putative compound names using different databases PlantCyc (http://www.plantcyc.org/), METabolite LINk (METLIN) (https://metlin.scripps.edu), KNApSAcK (http://kanaya.naist.jp/KNApSAcK/) and Kyoto encyclopedia genes and genomes (KEGG) (http://www.genome.jp/kegg/). The putatively identified metabolites were further confirmed based on: i) accurate mass match (with accurate mass error < 5 ppm) [6,35]; ii) fragmentation pattern match [35,38].

### Sample collection, RNA extraction, library preparation, Illumina sequencing, and data analysis

For transcriptome analysis, three alternate pairs of spikelets of three spikes per RIL were inoculated with *F*. *graminearum* spore suspension and water (control). Each treatment (resistant pathogen = RP, resistant mock = RM, susceptible pathogen = SP, susceptible mock = SM) consisted of three biological replicates. At 48 hpi, the three inoculated and three un-inoculated spikes were harvested, the rachis in inoculated region was harvested, and ground in liquid nitrogen using pre-chilled mortar and pestle. 100 mg of tissue samples were weighed in 2 ml sterilized micro-centrifuge tubes and the total RNA was extracted using Qiagen RNeasy Mini Kit (Qiagen, Germany). Total RNA was quantified using a NanoDrop Spectrophotometer ND-1000 (NanoDrop Technologies, Inc.) and its integrity was assessed using a 2100 Bioanalyzer (Agilent Technologies). Libraries were prepared from 250 ng of total RNA using the TruSeq stranded mRNA sample preparation kit (http://www.illumina.com/products/truseq_stranded_mrna_sample_prep_kit.html), as per the manufacturer’s recommendation. Using the Poly-A selection, mRNA molecules were separated and fragmented, followed by cDNA synthesis, ligation of adapters and cDNA fragments enrichment (PCR) (http://www.illumina.com/applications/sequencing/rna/mrna-seq.html). Libraries were quantified using the Quant-iT^™^ PicoGreen^®^ dsDNA Assay Kit (Life Technologies) and the Kapa Illumina GA with Revised Primers-SYBR Fast Universal kit (D-Mark). Average size fragment was determined using a 2100 Bioanalyzer (Agilent Technologies). Libraries were sequenced with an Illumina Hiseq 2000 sequencer, with 100 bp paired-end reads (http://support.illumina.com/sequencing/sequencing_instruments/hiseq_2000.html). The Illumina Hiseq paired end raw reads were quality checked using FastQC. The raw reads were processed using ABLT in-house program to filter adapters and low quality bases towards 3'-end. The raw reads obtained were aligned to the reference genome using TopHat [103]. Cufflinks package was used to assemble transcript, estimates their abundances, and tests for differential expression and regulation [104]. The Cuffdiff program in Cufflinks was used to identify differentially expressed transcripts. The transcripts that were not aligned to reference genome were assembled using *de novo* assembly [39]. The transcripts were differentially classified as up-regulated, down-regulated and neutrally regulated. The transcripts with FC = ±1 were considered as neutrally regulated transcripts. Two treatments were compared at a time: RP vs RM, and SP vs SM, respectively, and further the fold change (FC) was calculated taking into consideration FC coming from resistant and susceptible genotypes to identify up, down, neutrally regulated expression (RP/RM = FC1, SP/SM = FC2, FC = FC1/FC2). The transcripts that were only expressed in one of those treatments compared at a time were provided with FPKM values, and the FPKM values were compared in resistant and susceptible to denote significantly higher expression. To make it clear the transcripts that were detected up-regulated only in resistant or susceptible genotype may be down-regulated or neutrally in other, or may be not significant at *P* < 0.01, so are not detected in the contrasting genotype in RNA-seq data.

### Bioinformatics annotation and methods

The transcripts were annotated using Blast2go program against several databases such as non-redundant protein database, Swiss-Prot, KEGG, etc. for annotation and GO functional classification [39]. The transcripts with *P* < 0.01 and log_2_ FC ≥ 1 were further retained. All the transcripts that were set to threshold of *P* < 0.01 were crosschecked with the preliminary annotation for all treatments. Pathway analysis was done by using KAAS (http://www.genome.jp/tools/kaas/). *Arabidopsis thaliana* (thale cress) and *Oryza sativa japonica* (Japanese rice) (RefSeq) were taken as reference. The transcription factors (TFs) were predicted by PlantTFDB webserver [105]. We used the threshold determination as set by the server and the established criteria to identify the candidate, which were considered for predicted TFs and the binding sites. Furthermore, the predictions were used for interpreting the GO annotations.

### Expression analysis using quantitative Real-Time PCR (qRT-PCR)

The RNA extracted for transcriptome analysis (RNA-seq) was used for cDNA synthesis. The first strand was synthesized using Affinity Script qPCR cDNA Synthesis Kit (Agilent Technologies, Canada). The qRT-PCR was performed in a reaction volume of 10 μl consisting of 25 ng cDNA, 2 pmole of each primer, Qi-SYBR Green supermix (BioRad, Canada). The reactions were carried out in CF”X384TM Real-Time system (BioRad, ON, Canada). The relative transcript abundance in pathogen treated treatments compared to water (control) treatments were analyzed using 2^−ΔΔC^_T_ (C_T_ = cycle threshold) [100]. The transcripts, only those were expressed in pathogen treated treatments; RP>SP were analyzed. The relative transcript abundance was represented as FC values; whereas, in RNA-seq data the values are presented as log_2_FC. The primers used for qRT-PCR analysis were designed using NCBI primer blast software (S4 Table) [106].

## Supporting Information

S1 Fig Gene ontology classification of high fold change transcripts detected in R-RIL and S-RIL upon *F*. *graminearum* inoculation at 48 hpi. (TIF)

S1 Table High fold change RRI and RRC metabolites detected and putatively identified in rachis tissues of wheat RIL carrying resistant alleles of QTL-Fhb2 following *F*. *graminearum* and mock inoculation. (XLSX)

S2 Table The transcripts with higher expression values in R-RIL and S-RIL following *F*. *graminearum* and mock inoculation at 48 hpi. (XLSX)

S3 Table The list of all the genes localized within the QTL-Fhb2 region based on the available survey sequence available. The genes within two markers GWM-133 and GWM-644 flanking the QTL-Fhb2 region were taken into account. The list of genes with gene ID, chromosome localization and gene ontology is given.
(DOCX)

S4 Table List of primers used for qRT-PCR analysis. (DOCX)
