# Supplementary figures and images for: Integrated Metabolo-Transcriptomics Reveals Fusarium Head Blight Candidate Resistance Genes in Wheat QTL-Fhb2
Source: PLoS One. 2016 May 27;11(5):e0155851. doi: 10.1371/journal.pone.0155851 (PMC4883744; doi:10.1371/journal.pone.0155851)

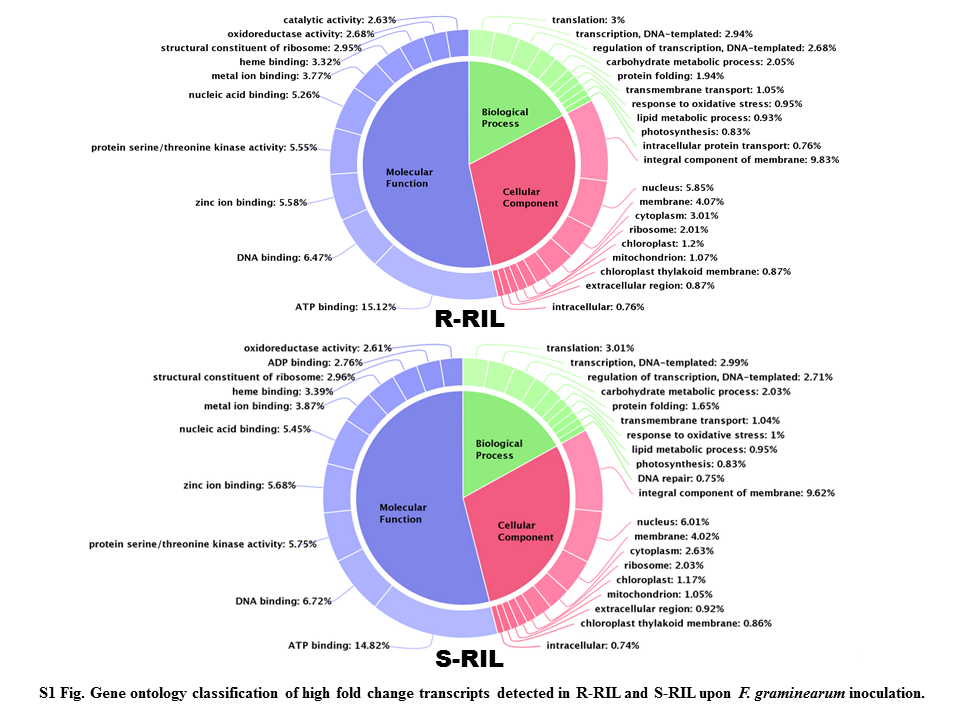

Supplement: S1 Fig — (TIF) [file pone.0155851.s001.TIF]
