# Supplementary material for: Integrated Metabolo-Transcriptomics Reveals Fusarium Head Blight Candidate Resistance Genes in Wheat QTL-Fhb2
Source: PLoS One. 2016 May 27;11(5):e0155851. doi: 10.1371/journal.pone.0155851 (PMC4883744; doi:10.1371/journal.pone.0155851)
Supplement: S3 Table — The genes within two markers GWM-133 and GWM-644 flanking the QTL-Fhb2 region were taken into account. The list of genes with gene ID, chromosome localization and gene ontology is given. (DOCX) [file pone.0155851.s004.docx]

S3 Table. The list of all the genes localized within the QTL-Fhb2 region based on the available survey sequence available. The genes within two markers GWM-133 and GWM-644 flanking the QTL-Fhb2 region were taken into account. The list of genes with gene ID, chromosome localization and gene ontology is given.

| **Gene Id** | **Locus** | **Putative Gene Name** |
| --- | --- | --- |
| Traes_6BS_6E43FD28C | 6B:98795903-98796251 | Zinc finger bed domain containing protein |
| Traes_6BS_D84ECBD67 | 6B:105401474-105408725 | uncharacterized protein |
| Traes_6BS_4EED05084 | IWGSC_CSS_6BS_scaff_3018346:2105-2720 | glutathione s-transferase 2 |
| Traes_6BS_BEE0205A4 | IWGSC_CSS_6BS_scaff_3022564:3575-4055 | retrotransposon expressed |
| Traes_6BS_BB64B9E1C | IWGSC_CSS_6BS_scaff_3025253:734-1019 | disease resistance rpp8-like protein 4 |
| Traes_6BS_D7B2695ED | IWGSC_CSS_6BS_scaff_3044824:6-2537 | Linalool synthase |
| Traes_6BS_C90105AAC | IWGSC_CSS_6BS_scaff_3042154:296-4412 | pentatricopeptide repeat-containing protein |
| Traes_6BS_1668BA98C | IWGSC_CSS_6BS_scaff_3034260:829-27467 | callose synthase 7-like |
| Traes_6BS_C44E33CC9 | IWGSC_CSS_6BS_scaff_3033579:933-3458 | Receptor-like serine/threonine-protein kinase NCRK |
| Traes_6BS_CC8E63D7F | 6B:101930446-101933974 | 4-coumarate: ligase |
| Traes_6BS_6477278C5 | 6B:115159074-115159556 | cinnamyl alcohol dehydrogenase |
| Traes_6BS_0B827F0AF | IWGSC_CSS_6BS_scaff_3022053:381-1517 | rna polymerase i-specific transcription initiation factor rrn3-like isoform x2 |
| Traes_6BS_77D8DE4BC | IWGSC_CSS_6BS_scaff_3034573:748-4359 | LRR receptor-like serine threonine-protein kinase |
| Traes_6BS_B577BF860 | 6B:107593782-107594882 | (+)-delta-cadinene synthase isozyme xc14 |
| Traes_6BS_C731E68C0 | 6B:113024876-113025227 | NA |
| Traes_6BS_5FDCAC5AC | IWGSC_CSS_6BS_scaff_3023594:4222-4498 | NA |
| Traes_6BS_1D0EF1E7F | 6B:98479975-98481045 | cytochrome p450 71D7 |
| Traes_6BS_89E8D55D0 | 6B:117324136-117332155 | carboxy-terminal kinesin protein |
| Traes_6BS_C9A6CC75D | IWGSC_CSS_6BS_scaff_3044780:1180-3851 | alpha-galactosidase |
| Traes_6BS_9D419E581 | IWGSC_CSS_6BS_scaff_3033571:347-3015 | f-box protein |
| Traes_6BS_BFAD78604 | IWGSC_CSS_6BS_scaff_3043129:1-1012 | Linalool synthase |
| Traes_6BS_56A7B216D | IWGSC_CSS_6BS_scaff_3029471:1852-4054 | cinnamyl alcohol dehydrogenase |
| Traes_6BS_67DF198CC | 6B:106644693-106648084 | protein strubbelig-receptor family 8-like |
| Traes_6BS_F74C5C315 | IWGSC_CSS_6BS_scaff_3042895:0-1009 | wall-associated receptor kinase 5 |
| Traes_6BS_80E76164B | IWGSC_CSS_6BS_scaff_3034647:9624-11788 | wall-associated receptor kinase 5-like |
| Traes_6BS_640931130 | IWGSC_CSS_6BS_scaff_3045210:4022-6487 | peptide transporter ptr3-a |
| Traes_6BS_2E20923DC | IWGSC_CSS_6BS_scaff_3021572:0-245 | LRR receptor-like serine threonine-protein kinase |
| Traes_6BS_D84ECBD67 | 6B:105401474-105408725 | predicted protein |
| Traes_6BS_67DF198CC | 6B:106644693-106648084 | protein strubbelig-receptor family 8-like |
| Traes_6BS_AA30AA645 | 6B:120407586-120414197 | Lrr receptor-like serine threonine-protein kinase |
| Traes_6BS_1D0EF1E7F | 6B:98479975-98481045 | cytochrome p450 71D7 |
| Traes_6BS_B9E883148 | 6B:100433175-100434055 | zinc-binding protein |
| Traes_6BS_478D6230A | 6B:100982962-100983390 | defensin-like protein 6 |
| Traes_6BS_99EC5A760 | 6B:113965810-113970403 | bri1-kd interacting protein |
| Traes_6BS_D6C182082 | 6B:117328299-117332155 | Kinesin-4 |
| Traes_6BS_85577CA32 | 6B:121546584-121547073 | udp-glycosyltransferase 71c4 |
| Traes_6BS_EAEA12107 | IWGSC_CSS_6BS_scaff_3015607:354-639 | glutathione s-transferase t3-like |
| Traes_6BS_DF0897773 | IWGSC_CSS_6BS_scaff_3015877:3079-6263 | protein argonaute pnh1-like |
| Traes_6BS_1155DB1BE | IWGSC_CSS_6BS_scaff_3026382:6300-6785 | cox viia-like protein |
| Traes_6BS_A17151647 | IWGSC_CSS_6BS_scaff_3028132:4199-4460 | histone deacetylase hdt2 |
| Traes_6BS_C44E33CC9 | IWGSC_CSS_6BS_scaff_3033579:933-3458 | receptor protein kinase perk1 |
| Traes_6BS_935865CEA | IWGSC_CSS_6BS_scaff_3034551:3-1168 | methyl- -binding domain-containing protein 13 |
| Traes_6BS_FA7E5AC2D | IWGSC_CSS_6BS_scaff_3038292:4983-7753 | tata box-binding protein-associated factor rna polymerase i subunit b isoform x1 |
| Traes_6BS_62EDA03FF | IWGSC_CSS_6BS_scaff_3040207:915-10974 | beach domain-containing protein lvsc-like |
| Traes_6BS_EA1DF4148 | IWGSC_CSS_6BS_scaff_3043601:2-3699 | start domain-containing protein |
| Traes_6BS_9BDAEC739 | IWGSC_CSS_6BS_scaff_3045102:1456-10204 | ---NA--- |
| Traes_6BS_6C2DE4A9E | IWGSC_CSS_6BS_scaff_3033646:1225-2044 | hydroquinone glucosyltransferase-like |
| Traes_6BS_CA8516D45 | IWGSC_CSS_6BS_scaff_3031055:4-2387 | protein disulfide isomerase-like 1-4 |
| Traes_6BS_6B28A9694 | IWGSC_CSS_6BS_scaff_3041371:175-2043 | autophagy protein 5 |
| Traes_6BS_62EDA03FF | IWGSC_CSS_6BS_scaff_3040207:841-10974 | beach domain-containing protein lvsc-like |
| Traes_6BS_3D67A0818 | 6B:100825660-100827460 | NA |
| Traes_6BS_560511373 | 6B:115995453-115998073 | protein wvd2-like 1 |
| Traes_6BS_935865CEA | IWGSC_CSS_6BS_scaff_3034551:3-1168 | methyl- -binding domain-containing protein 13 |
| Traes_6BS_B59D85BB7 | IWGSC_CSS_6BS_scaff_3042796:188-729 | NA |
| Traes_6BS_698D9AFAC | IWGSC_CSS_6BS_scaff_3040376:3397-4978 | pentatricopeptide repeat-containing protein at4g14850 |
| Traes_6BS_9BE4E2745 | 6B:117242506-117247729 | ac078948_18 serine protease |
| Traes_6BS_62EDA03FF | IWGSC_CSS_6BS_scaff_3040207:841-10974 | beach domain-containing protein lvsc-like |
| Traes_6BS_C08C1D737 | 6B:117316588-117320397 | sugar transporter type 2a protein |
| Traes_6BS_C44E33CC9 | IWGSC_CSS_6BS_scaff_3033579:933-3458 | receptor-like serine threonine-protein kinase ncrk |
| Traes_6BS_935865CEA | IWGSC_CSS_6BS_scaff_3034551:3-1168 | methyl- -binding domain-containing protein 13 |
| Traes_6BS_4EA352A7A | IWGSC_CSS_6BS_scaff_3043365:17-1651 | gata transcription factor 22 |
| Traes_6BS_99EC5A760 | 6B:113965810-113970425 | zinc finger ccch domain-containing protein 13 |
| Traes_6BS_9BDAEC739 | IWGSC_CSS_6BS_scaff_3045102:1460-12670 | ---NA--- |
| Traes_6BS_E323ECAC1 | IWGSC_CSS_6BS_scaff_3044944:279-845 | heat stress transcription factor a-9 |
| Traes_6BS_C90105AAC | IWGSC_CSS_6BS_scaff_3042154:333-4412 | protein low psii accumulation chloroplastic |
| Traes_6BS_19F006833 | IWGSC_CSS_6BS_scaff_3041696:0-2933 | hypothetical protein F775_11456 |
