# Supplementary material for: Integrated Metabolo-Transcriptomics Reveals Fusarium Head Blight Candidate Resistance Genes in Wheat QTL-Fhb2
Source: PLoS One. 2016 May 27;11(5):e0155851. doi: 10.1371/journal.pone.0155851 (PMC4883744; doi:10.1371/journal.pone.0155851)
Supplement: S4 Table — (DOCX) [file pone.0155851.s005.docx]

S4 Table. List of primers used for qRT-PCR analysis.

| Gene | Forward Primer | Reverse Primer |
| --- | --- | --- |
| 4 Coumarate CoA-ligase | GCACCCGGCGTGGAAT | GAGGCCGATGTCTCCGGT |
| bHlH041 transcription factor | ACCAGACGAAGCAACCCTTCAA | TCAAAGTTCGTCGGGCGACGG |
| ABC transporter-4 | TTGGCAACTCTTGGGGTTTCG | GCATCATGCCCTCATCGCTAC |
| Glutathione S-transferase | CTCTGTGTTTCAGCTGGTAGCCC | CGCTACGTCGACGAGCCCTA |
| Chitinase 1 | CTGCTCACCCAGCTCAAGG | TTCCTCCACAGAGCCTGGTA |
| Chalcone Synthase | CATCGGGCAGAACGTGGA | CCCTGATAACGGAGACGGAAG |
| Phenylammonia lyase | CCCACTCTACCGATTCGTGC | ATGTGCTTGCCCTGGTTCAT |
| MYB-4 | CCAAGAAGCCTGTGGCTGTA | GATGGCATCGAGGAATCGGT |
